# System Performance Analysis for Trimmable Horizontal Stabilizer Actuator Focusing on Nonlinear Effects: Based on Incremental Modelling and Parameter Identification Methodology

**DOI:** 10.3390/s21196464

**Published:** 2021-09-28

**Authors:** Wensen Zhang, Jian Fu, Yongling Fu, Jinlin Zhou, Xudong Han

**Affiliations:** 1 School of Mechanical Engineering and Automation, Beihang University, Beijing 100191, China; wensenzhang@buaa.edu.cn (W.Z.); fuyongling@buaa.edu.cn (Y.F.); jlzhou@buaa.edu.cn (J.Z.); 18813094151@163.com (X.H.); 2 Laboratory of Aerospace Servo Actuation and Transmission, Beihang University, Beijing 100191, China

**Keywords:** trimmable horizontal stabilizer actuator, nonlinear effect, system performance, incremental methodology, parameter identification, multi-level modelling

## Abstract

With the development of more/all electric aircraft, replacement of the traditional hydraulic servo actuator (HSA) with an electromechanical actuator (EMA) is becoming increasingly attractive in the aerospace field. This paper takes an EMA for a trimmable horizontal stabilizer as an example and focuses on how to establish a system model with an appropriate level of complexity to support the model-based system engineering (MBSE) approach. To distinguish the nonlinear effects that dominate the required system performance, an incremental approach is proposed to progressively introduce individual nonlinear effects into models with different complexity levels. Considering the special design and working principle of the mechanical power transmission function for this actuator, the nonlinear dynamics, including friction and backlash from the no-back mechanism, and the nonlinear compliance effect from the mechanical load path are mainly taken into consideration. The modelling principles for each effect are addressed in detail and the parameter identification method is utilized to model these nonlinear effects realistically. Finally, the responses from each model and experimental results are compared to analyze and verify how each individual nonlinearity affects the system’s performance.

## 1. Introduction

With the development of partly/fully electric aircraft, the application of power-by-wire (PBW) actuations has received significant interest [1,2]. The PBW technology achieves the replacement of central hydraulic systems, including distribution networks (pipes and fluid) and valve blocks, by electric systems, which is advantageous for power management, integration, and maintenance [3,4]. Two categories of PBW actuators, which are named the electro-hydrostatic actuator (EHA) and EMA, are employed in commercial civil aircrafts. EHAs still use hydraulics locally, whereas EMAs remove both central and local hydraulic circuits by transmitting motor power to the load through mechanical components [5,6]. Although EMAs are not yet mature enough for primary flight control because of their jamming probability, there appears to be greater potential in actuation systems for a trimmable horizontal stabilizer (THS), which is a less safety-critical secondary flight control component [7,8,9]. 

The introduction of electrical actuation for flight control raises new challenges, including power sizing, back-drivability, load rejecting, cushioning and damping, response to failures, and heat rejections [3]. In such a situation, the MBSE approach provides engineers with efficient means to address all these critical issues as a whole, and it is an unquestionably ideal solution [10,11]. This simulation-driven approach requires establishing meticulous component models with realistic physical effects and developing a system-level virtual test. However, for numerical implementation consideration and time-saving purposes, the best model must possess an appropriate complexity level. For this purpose, it is important to analyze how the nonlinear dynamics impact the system performance. Then, the less important nonlinear effects can be neglected or only the effects that dominate the required system performance can be studied. Additionally, this study can serve for further control algorithm optimization and provide important experience in power sizing and thermal balance [12].

However, to execute this process, there still remain two major challenges. The first one refers to the issue of realistically modelling different categories of nonlinear effects, such as backlash, preload, and friction. A recommended method is to attempt to combine the knowledge models (from scientific points) and representation models (from actual or experimental tests). The next challenge mainly concerns how to individually evaluate physical effects. With regard to an electromechanical system with only linear or simple nonlinear effects, the known methods, such as system principle analysis or automatic control theory, are sufficient to solve this problem with higher efficiency and lower cost. However, when the physical effects are mixed and complex, the traditional approaches are no longer the superior option. A practical approach is to adopt an incremental methodology during the model development process. The model complexity can be progressively increased based on whether each nonlinear effect is considered. This incremental methodology can ensure standard interfaces and replaceability in multi-level modelling. Additionally, when the multi-domain effects are concerned, the bond graph can be introduced as the modelling language [13]. 

The rest of this article is organized as follows: Section 2 presents system working principle analysis and conducts the multi-level modelling process of the THSA system following the incremental and parameter identification methodology. Then, in Section 3, the system performance differences between simulation and experimental results are compared to verify how individual nonlinearities affect system performance. Finally, the Conclusions summarize the main achievements of this study.

## 2. Materials and Methods

### 2.1. System Description

**Figure 1:**
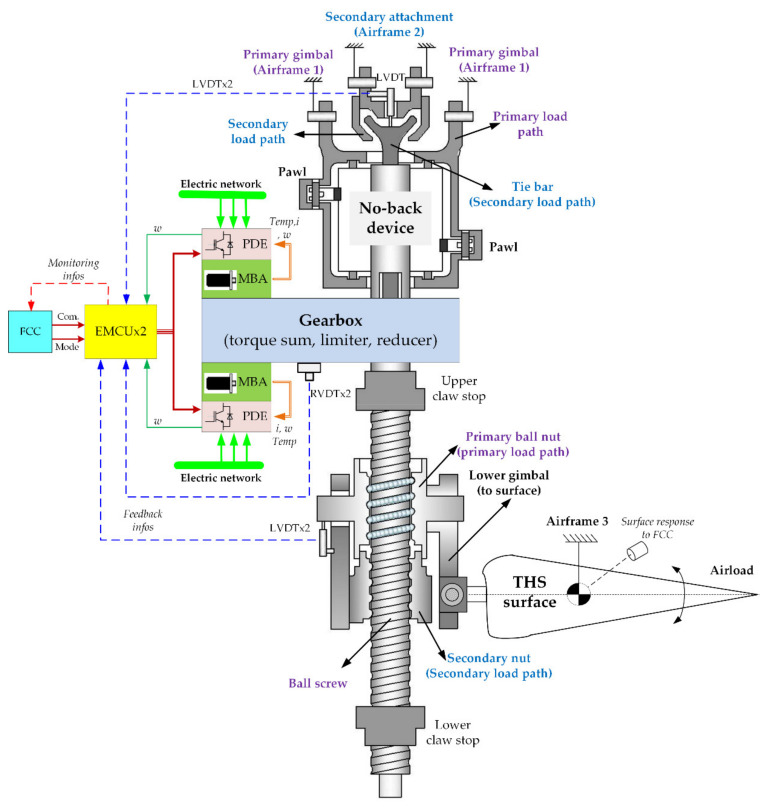
Architecture of THSA system.

This research deals with a trimmable horizontal stabilizer actuator (THSA) for aircraft, which mainly functions for auxiliary pitch control [14,15]. As shown schematically in Figure 1, the adopted actuation architecture comprises the following components:

Electrical motor control unit (EMCU): Based on the motor speed feedback and trim speed command from the flight control computer (FCC), velocity loop regulation and current command generation for power drive electronics (PDE) are executed in the EMCU. Meanwhile, according to signals sampled from sensors, such as the linear variable differential transducer (LVDT), rotary variable differential transducer (RVDT), etc., the EMCU is in charge of fault diagnosis with specific strategies [16]. The diagnosis results interact with the FCC to disable the defective actuation channel [17].

PDE: The power flow between the electrical supply and the motor, as well as the current loop regulation, is conducted. 

Motor and brake assembly (MBA): THSA involves a primary MBA and a backup MBA, which are configured in torque summing mode. In principle, the backup MBA operating in passive mode functionally offers no resistance to the primary MBA.

Gearbox: In addition to the fundamental velocity reduction function, a torque limiter is internally integrated inside, which is responsible for motor protection under overload situations. Dual RVDTs, installed in the gearbox and backing up each other, feedback the current THS surface position to the EMCU and FCC for monitoring and fault processing.

Screw-nut mechanism: The rotary motion is translated into linear motion via a ball screw–nut mechanism. The secondary nut works as a backup and remains unloaded in normal mode.

Mechanical load path: The mechanical load path enables the transmission of an external axial load to the airframe. In addition, dual mechanical load paths, the respective components of which are illustrated and distinguished with different colors in Figure 1, are utilized for mechanical redundancy.

No-back device: The fundamental function of a no-back mechanism is to mechanically lock the surface position [17]. The detailed compositions and working principles will be addressed in detail.

### 2.2. Incremental Modelling

Usually, the basic model for a component can be easily determined from its function and property. However, in order to successfully establish complex models, further modelling principle analysis is inevitable, which can locate where the nonlinear effect functions and conduct accurate mathematical description. Based on actual needs, knowledge models or representation models (identified from actual or experimental tests) can be used to evaluate the nonlinear effects.

#### 2.2.1. Modelling Procedure

**Figure 2:**
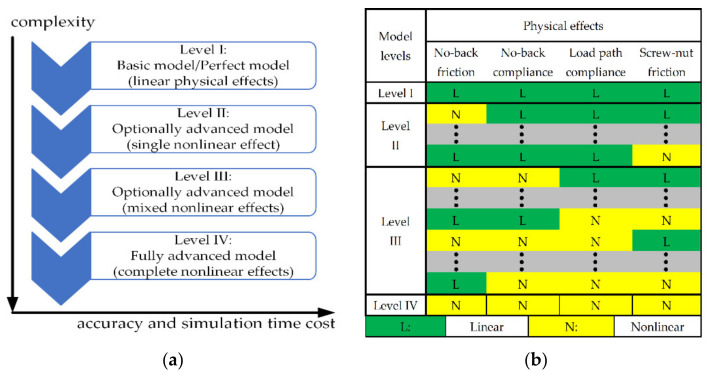
Multi-level modelling with incremental methodology. (**a**) Model complexity versus accuracy and simulation time cost; (**b**) Potential model matrixes with different levels for THSA.

The modelling procedure requires prior planning in order to follow the incremental methodology. As depicted in Figure 2a, different generic levels of model packages can be defined according to the different complexities of physical effects. The levels are organized from the simplest to the most complex, with the associated impacts on accuracy and simulation time cost. For a level I model, all the physical effects are linear, whereas a level IV model is fully advanced by describing complete nonlinear effects. Both level II and level III models are optionally advanced, and the difference is dependent on whether single or mixed nonlinear effects are introduced in a specific model.

From the above system description of THSA, compared to a conventional EMA [18], the major differences result from the geometry and configuration of components for the mechanical power transmission function, such as the mechanical load path and no-back mechanism. Meanwhile, the physical effect for the electrical motor has been addressed in early research [19] and the high-frequency dynamic caused by PDE is less critical for a generic position/speed servo system [20]. With regard to mechanical power transmission components, both friction and compliance effect parameters determine the system performance. The former impact the transient response and possibly cause the limited cycle phenomenon, whereas the latter affect the load stiffness and lead to oscillation during position tracking and frequency response. Hence, the main considered effects include backlash and preload in the mechanical load path, mixed nonlinear effects from the no-back mechanism, and screw–nut mechanism friction. The potential model matrixes with different levels for THSA are shown in Figure 2b.

#### 2.2.2. Modelling Hypothesis

In order to propose more suitable models for friction and compliance effects with backlash or preload, the following hypotheses are considered:

**Hypothesis** **1.** *The primary source of no-back friction torque results from the inserted friction disks.*

**Hypothesis** **2.** *The variable stiffness phenomenon is neglected when two components gradually eliminate the backlash and achieve entire contact deflection, i.e., the stiffness nonlinearity is evaluated by the sign function.*

**Hypothesis** **3.** *For the no-back mechanism, the backlash phenomenon depends on the direction of rotation of the screw and the position of each tooth of the ratchet wheel determines which one will eventually mesh with the pawl. For simplification, the contact stiffness is considered constant, as the number of tooth and the direction vary during the meshing [21].*

#### 2.2.3. Ball Screw–Nut Mechanism

The basic function of the ball screw–nut mechanism is to translate rotary motion into linear motion. Its internal compliance effect is merged into a mechanical load path; for this reason, only the friction effect is taken into consideration here.

1. Basic model

Translation from rotational into linear motion occurs at the screw–nut coupling and the sliding/rolling frictions arising at the contact between the threads are characterized by a screw pitch *p* and efficiency coefficient *η*, respectively. The output force and linear speed can be defined as:

(1) $$\left\{\begin{array}{l} F_{s}=T_{s}*\mathrm{TF}*\eta \\ v_{s}=\omega_{s}/\mathrm{TF} \end{array}\right.\;\mathrm{TF}=\frac{2\pi }{p}\;$$

2. Advanced model

For simplification, the friction effect will be characterized consistently with the basic model for two reasons. Firstly, the friction inside the screw–nut mechanism is the result of the combination of operating quadrant (aiding or opposing load), normal force and relative nut–screw velocity, which is difficult to evaluate without sufficient experimental data [22]. Secondly, the application of the no-back mechanism dominates the total friction torque, which is why the friction effect for the screw–nut mechanism can be approximately simplified.

#### 2.2.4. Mechanical Load Path

The mechanical load path enables the transmission of an external axial load to the airframe. Obtaining its realistic servoelastic behavior is particularly necessary, because the compliance effect may significantly impact the dynamic performance and the service life of the actuator.

1. Overall analysis

The respective components of dual load paths are illustrated in Figure 1. The secondary load path works as mechanical redundancy, by sharing the external load in the event of primary load path failure. In normal operation, the initial assembly clearances guarantee that no force can be transmitted via the secondary load path. For this reason, the principle analysis can be simplified if fault-tolerant function research is not conducted temporarily.

**Figure 3:**
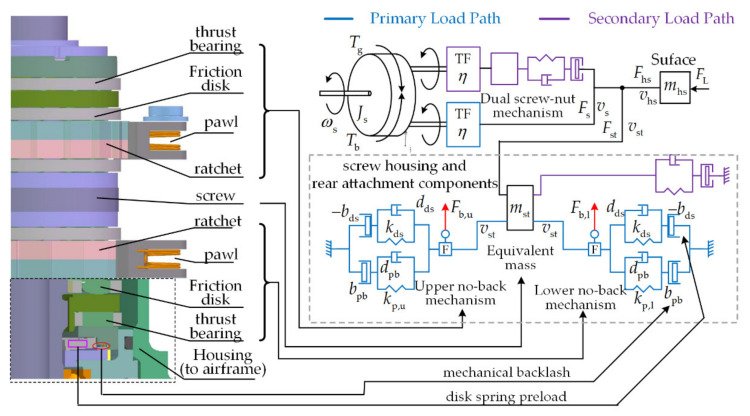
Working principle diagram of mechanical load path.

A working principle diagram of the mechanical load path components is provided in Figure 3. The power input is the torque *T*_g_ and screw velocity *ω*_s_ from the gearbox, whereas the power output is the trimming speed of the surface and drive force to balance the aerodynamic force *F*_L_. Torque input *T*_g_ can be decomposed into several components: inertia torque loss *J*_s_, friction torque loss *T*_b_ from the no-back mechanism, and screw driving torque *T*_s_. 

The transformation of rotary motion to translation motion is accomplished via the ball screw–nut mechanism. The control surface load is transmitted via the screw–nut mechanism and exerts an axial load to the screw. This axial load eventually is supported by the screw housing and rear attachment components.

2. Basic model

In addition, the compliance effect existing in joints between the surface and actuator, as well as attachments between the screw housing/body and airframe, is considered ideal, i.e., no backlash or preload deflection. Consequently, the drive force and speed of the surface can be derived as:

(2) $$\left\{\begin{array}{c} F_{\mathrm{st}}=F_{s}\\ v_{\mathrm{hs}}=v_{s} \end{array}\right.$$

3. Advanced model

The components of the screw housing and rear attachment are illustrated in Figure 3. Since the separate modelling of its individual compliance effect is difficult, modelling has been conducted at the global level, by merging the whole compliance effect into lumped stiffness and damper rating existing inside the screw housing and rear attachment components.

Based on the above geometry investigation of the no-back mechanism components in Figure 3, the tensile or compressive force exerted on the screw can be transmitted via two parallel paths. Moreover, each can be introduced as a compliance model, as displayed in Figure 3. The first one corresponds to a preload disk spring to ensure that both the upper and lower no-back mechanism components are tightly engaged, whereas the other corresponds to a spring-damper element with purposely reserved backlash.

For convenience, the backlash or preload effect can be defined by a proposed single parameter *x*_0_ with positive or negative signs, respectively. For a no-back mechanism with bilateral backlash *b*_pb_ and preload -*b*_ds_, the total transmitted force from screw to airframe is:

(3) $$F_{\mathrm{st}}=F_{b,l}+F_{b,u}=\left\{\begin{array}{ll} k_{p,u}(x_{\mathrm{st}}-b_{\mathrm{pb}})+2k_{\mathrm{ds}}x_{\mathrm{st}}+d_{p}{\dot{x}}_{\mathrm{st}} & x_{\mathrm{st}}\geq b_{\mathrm{pb}}\\ 2k_{\mathrm{ds}}x_{\mathrm{st}} & -b_{\mathrm{pb}}<\;x_{\mathrm{st}}<b_{\mathrm{pb}}\\ k_{p,l}(x_{\mathrm{st}}+b_{\mathrm{pb}})+2k_{\mathrm{ds}}x_{\mathrm{st}}+d_{p}{\dot{x}}_{\mathrm{st}} & x_{\mathrm{st}}\leq -b_{\mathrm{pb}} \end{array}\right.$$

where *x*_st_ is the additional linear displacement caused by the compliance effect of the load path between the screw housing and rear attachment components and the airframe; *k*_p,l_, *k*_p,u_ and *k*_ds_ are the equivalent no-back stiffness and stiffness of the preload disk spring; *d*_p_ is the contact damper rating; *F*_b,l_ and *F*_b,u_ are, respectively, the transmitted force by the lower and upper no-back mechanism. With the addressed modelling hypothesis, the constant parameters *k*_p,l_ and *k*_p,u_ are denoted as equivalent no-back stiffness for contact, whereas the stiffness is imposed to none for free play.

Considering the compliance effect of the load path, the surface speed is modified as:

(4) $$v_{\mathrm{hs}}=v_{s}+{\dot{x}}_{\mathrm{st}}$$

The stiffness parameters *k*_p,l_, *k*_p,u_, *k*_ds_ and backlash parameter *b*_pb_ must be identified by utilizing the widely used particle swarm optimization (PSO) algorithm. The load force *F*_st_ and surface displacement *x*_st_, which are accessible from the force sensor and linear displacement sensor installed on the nut, are the data input for this identification model from Equation (3). Each particle represents a different set of parameters (*k*_p,l_, *k*_p,u_, *k*_ds_, *b*_pb_) and the minimum identification error for load force *F*_st_ is regarded as the optimization objective. These particles are conceptual entities that travel through the multidimensional search space. At any particular time, each particle has a position and a velocity. The position vector of a particle represents a trial solution to the search problem. This search process will continue until the global best solution is acquired [23]. The step by step procedures have been described in detail in [24].

**Figure 4:**
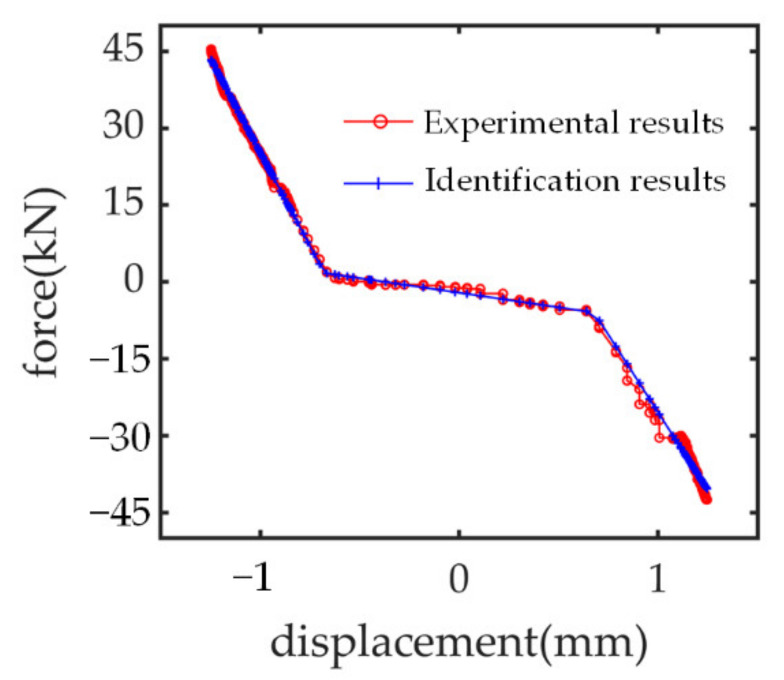
Stiffness identification with PSO algorithm.

The identification results and the comparison between measured force and identified force versus displacement are, respectively, plotted in Table 1 and Figure 4.

#### 2.2.5. No-Back Mechanism

Attributed to the no-back mechanism, the surface position can be mechanically locked and the power transmission chain is forced to be irreversible, which mainly rely on two pawl and ratchet wheel assemblies, as well as the friction disks inserted between its components, as in Figure 3. The friction torques generated from friction disks, which vary remarkably under opposing or aiding load, are worthy of careful consideration. Moreover, the circumferential clearance between the pawl and ratchet wheel alters dynamically, which directly affects the actuator uncontrolled displacement and compliance effect of the no-back mechanism, requiring adequate attention.

1. Overall Analysis of Modelling Principle

**Figure 5:**
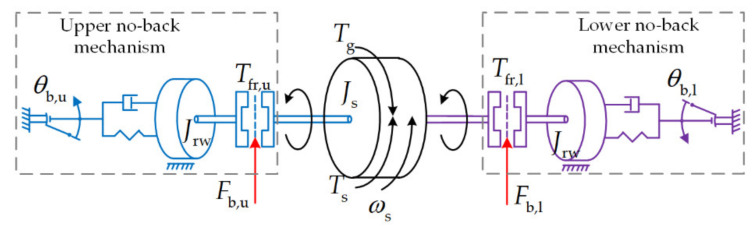
Schematic diagram of no-back mechanism.

To realize the bidirectional irreversibility function, the no-back pawls block the rotation of their respective ratchet wheels relative to the actuator body, each operating for a different rotation direction, as is shown in Figure 3. The complete working principle model of the no-back mechanism is schematically shown in Figure 5. If, for example, the actuator is considered inactive, the angular velocity is zero (*ω*_s_ = 0), and a compressive load (positive sign) *F*_st_ is transferred to the screw when a load of the same direction is exerted on the nut. Moreover, two virtual force sensors are inserted to acquire the real-time impact forces *F*_b,u_ and *F*_b,l_ of the bidirectional no-back assembly components:

- the upper brake mechanism is axially tightly engaged and essentially no friction torque *T*_fr,l_ is developed from the lower brake mechanism in either direction, which is because the minor impact force *F*_b,l_ remains between its components;
- the upper ratchet wheel is blocked by its pawl to avoid the screw revolving due to the axial force exerted by the load on the nut;
- the load torque *T*_L_ generated on the screw by the external load is balanced by the stick friction torque *T*_fr,u_ produced by the upper friction disks.

For one and the same direction of load force, if the actuator is active, it must move the nut upward, corresponding to a negative sign of screw velocity *ω*_s_. Therefore, the movement has the same direction as the force exerted by the load on the actuator: the load aids this. The actuator must develop a driving torque *T*_s_ on the screw in order to override the breakaway friction torque *T*_fr,u_ produced from the upper friction disk. Functionally, essentially no friction torque is developed from lower brake mechanism, since the lower ratchet wheel is permitted to be freewheeling in the opposite direction. 

On the contrary, if the actuator must move the nut downward, corresponding to a positive sign of *ω*_s_ (the load is opposing), the ratchet wheels for the lower brake mechanism and upper brake mechanism are blocked and permitted to be freewheeling, respectively, which implies that operation states for both ratchet wheels are inversed. Consequently, the friction torque *T*_fr,l_ generated from the lower friction disk is overridden, whereas essentially no friction torque *T*_fr,u_ is developed from the upper brake mechanism. The amplitude of *T*_fr,l_ is finite since the impact force *F*_b,l_ is limited within the spring preload and grows smaller with the increase in compressive load.

2. Basic model

The basic model is to introduce the friction torque *T*_b_ generated from the no-back mechanism as a coulomb torque, which is the combination of the upper no-back mechanism *T*_fr,u_ and lower no-back mechanism *T*_fr,l_. To guarantee the no-back function of THSA, the amplitude of this coulomb torque is imposed to:

(5) $$T_{b}=F_{L}/\mathrm{TF}$$

3. Advanced model

(1) Realistic friction

Friction loss is a very complex phenomenon that is highly dependent on velocity, external load, and temperature [12]. The suggested model to evaluate the friction in this situation is:

(6) Tf=(μc+(μs−μc)e(−(ωs/ωst)^2))|F|sgn(ωs)+fsωs

where *μ*_s_ and *μ*_c_ are the stick and coulomb friction coefficient, respectively; *ω*_st_ is the stribeck reference velocity; *F* is the normal impact force; *f*_s_ is the viscous friction coefficient.

**Figure 6:**
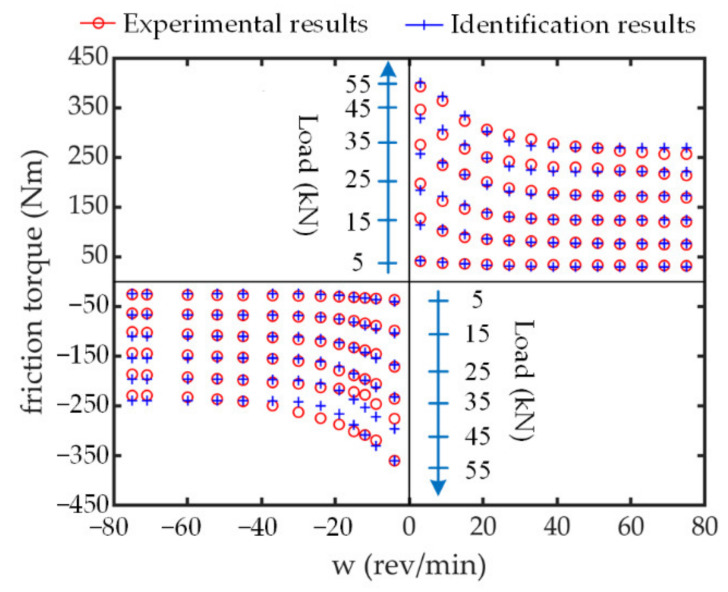
No-back friction identification with PSO algorithm.

It is emphasized that this proposed model includes the impact force transmitted from the external load, this significant effect being generally ignored in the modelling of mechanical transmission devices. Based on the modelling approach in Equation (6), the friction modelling can be accomplished with the following parameter identification process. As discussed above, the friction torque that requires identification is generated from the friction disk. A dedicated test rig is designed for this purpose [25]. This test rig can drive the friction disk rotating at a controlled angular velocity and a load simulator can exert load force with random desired amplitudes. By varying the screw velocity from 3 rev/min to 75 rev/min (corresponding to motor nominal velocity 5000 rev/min) and the load force between 0 kN and 55 kN, which covers most of the operating domain for THSA, the friction torque is recorded from high-precision sensors. Adopting the same algorithm in Section 2.2.3, the identification results and the comparison between measured friction and identified friction are, respectively, shown in Figure 6 and Table 2. It can be seen that the viscous friction is nonexistent. Further investigations show that the regular viscous friction may diminish with the speed increasing due to the lubricant temperature rise [22]. Meanwhile, nonnegligible differences exist between the friction parameters for bipolar rotating directions, which may be caused by the skew angle of the rollers [26]. 

(2) Backlash effect

As addressed above, each ratchet wheel exists in two operation states, i.e., freewheeling and blocked. When the screw velocity changes to the opposite direction, the state of each ratchet wheel inverses functionally. However, as shown in Figure 5, due to the existence of a circumferential angle between adjacent ratchet teeth, a circumferential clearance must be eliminated before the ratchet wheel and pawl achieve active contact. More importantly, this circumferential clearance is dependent on the immediate position of the ratchet wheel. Attributed to strain gauges arranged on the housing of pawls, a strain signal will cause a sudden variation, when the ratchet wheel is blocked by the pawl. Consequently, the immediate circumferential clearance can be calculated according to the screw angular displacement difference from the velocity direction inverse clock to the block occurrence clock.

### 2.3. Model Selection and Test Rig Structure

The model matrixes in Figure 2 have provided many model implementation options. However, selecting these models, which can progressively introduce all nonlinear effects, is sufficient. In addition, a test rig for THSA with a proper structure is necessary for experiment verification.

#### 2.3.1. Model Selection

To demonstrate how different nonlinear dynamics affect system performance, the critical components in the proposed THSA model should be packed separately to modify its complexity levels incrementally. These levels must include the same ports to ensure that they are independent and replaceable. Since the nonlinear effects that we focus on are friction and backlash in the no-back mechanism and the compliance effect in the mechanical load path, four models with progressively complex levels, which are part of the alternatives of the model matrix in Figure 2b, are utilized: (a) a basic model, (b) an optional advanced model with only friction from the no-back mechanism, (c) an optional advanced model with friction and backlash from the no-back mechanism, and (d) a fully advanced model. These models can be easily built in the AMESim or MATLAB/Simulink platform following the schematic diagrams in Section 2.2. The potentially evaluated parameters are displayed in Table 3.

#### 2.3.2. Test Rig Structure

**Figure 7:**
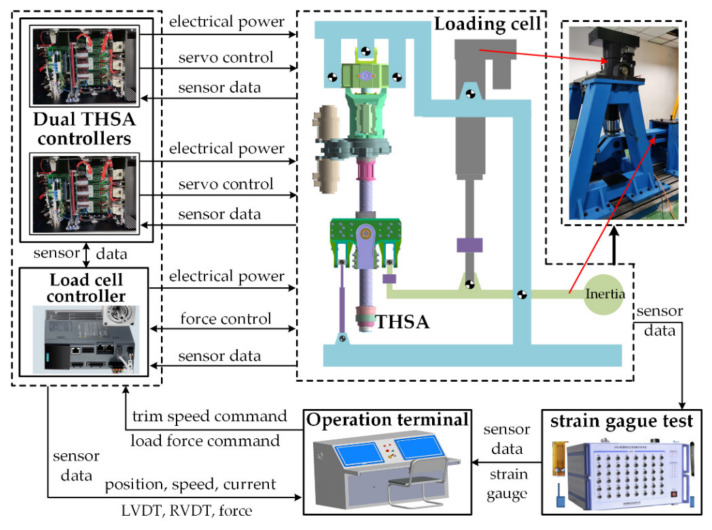
Test rig for THSA.

As shown in Figure 7, a test rig for THSA is built for experiment verification. This test rig enables us to perform position/speed servo control of THSA. In addition, a load simulator is used to exert a random, desired aerodynamic force for THSA. The strain gauges are arranged on the housing for stress measurement. The trim speed control and load control are executed by corresponding controllers, based on the command signals from the operation terminal and sensor data. All the experimental results are available in the operation terminal for operators. Moreover, the data acquired from the force sensor installed on the test rig are utilized as aerodynamic force simulation input to improve the reliability of the simulation results.

## 3. Simulation and Experimental Results Analysis

To analyze how nonlinear dynamics, including friction parameters and compliance parameters, affect system performance, it is necessary to compare the system performance differences between the simulation and experimental results, when each individual nonlinear dynamic is progressively introduced. In addition, model levels should be flexible to match various purposes. 

### 3.1. Hybrid Nonlinear Effects of No-Back Mechanism

The impact on system performance of no-back friction parameters is established by comparing the speed and current response of models with different level (b), (c) and experimental results. The effect of rotary backlash parameters on system performance is evaluated by comparing the speed response of different level models (b), (c) and the experimental results. For these purposes, a speed command with four periods and a positive ramp aerodynamic compressive force (amplitude 29 kN, slope 58 kN/s) at time 1 s is first introduced. The bipolar trapezoidal trim speed commands with an amplitude 15 mm/s and acceleration 30 mm/s^2 are, respectively, applied at time 1 s and time 6 s. Moreover, the actuator is disabled during the rest periods, i.e., no active torque output for the motors. 

**Figure 8:**
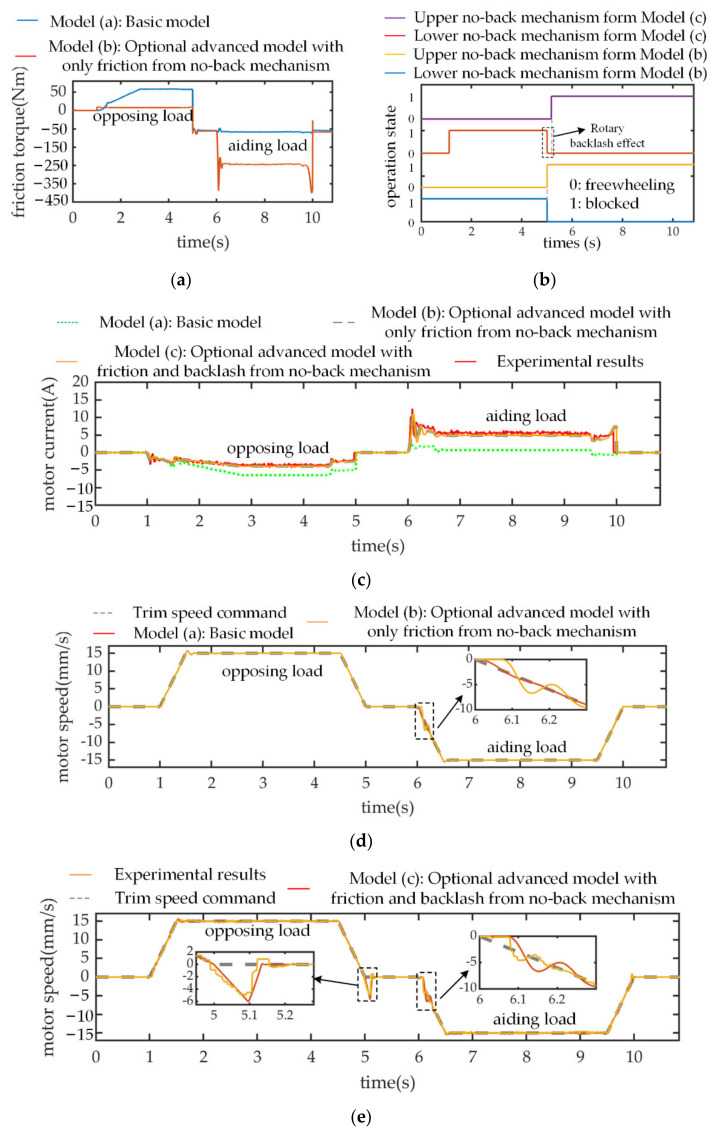
Simulation responses and experimental results under bipolar speed command and ramp load. (**a**) No-back friction torque; (**b**) Operation state of pawl and ratchet wheel; (**c**) Motor current response; (**d**) Trim speed response from models (**a**,**b**); (**e**) Motor speed response from model (**c**) and experimental result.

Based on the friction analysis in Section 2.2.4, when the THSA operates from positive speed to negative speed, the load transforms from an opposing state into an aiding state, and the ratchet wheel from the lower and upper no-back mechanism is, respectively, blocked (Figure 8b). Consequently, the primary source of no-back friction torque varies from the lower friction disk to upper friction disk. As displayed in Figure 8a, unlike model (a), these two friction torques show a great amplitude difference in model (b). The reason is that the compressive aerodynamic load is mostly transmitted by the upper no-back mechanism components and the friction amplitude is primarily determined by normal impact force. This explains why the motor provides a smaller current under opposing load conditions but a larger current (approximately 3 times larger) under aiding load conditions, which is a common phenomenon observed in the current simulation results in model (b) and the experimental results (Figure 8c). In addition, the no-back friction causes a speed perturbation (around 0.1 s) in model (b) (Figure 8d) at the activation stage due to the sudden increase in no-back friction torque under the aiding load, which is similarly observed in the experimental results (Figure 8e).

The effect of rotary backlash on system performance is established by comparing the speed responses of different level models (b,c) and experimental results, and the working principle analysis of the no-back mechanism in Section 2.2.4 will be critically considered. As addressed above, during the second and last periods of the speed command signal, THSA receives the trim stop command, and the motor is electrically disabled. Consequently, under compressive or tensile aerodynamic load, the surface position is mechanically locked via the upper or lower no-back mechanism, respectively. In this compressive load situation, when THSA operates at positive speed, the ratchet wheel from the upper no-back mechanism is permitted to be freewheeling and the ratchet wheel from the lower no-back mechanism is blocked. With the introduction of clearance in model (c), while entering the first trim stop stage (around 4.95 s), the screw will be back-driven to eliminate the backlash between the pawl and ratchet wheel, before the ratchet wheel is blocked. This back-driven process causes a delay to occur in the ‘block’ state signal for the upper no-back mechanism, as seen when comparing the operation state simulation results in Figure 8b. Meanwhile, this back-driven phenomenon is consistent with the experimental result in Figure 8e, which verifies the analysis of the backlash effect. In addition, the maximum back-driven speed can approximately reach 40 percent of the command speed amplitude. When THSA operates at negative speed and second trim stop stage, the operation state for the complete no-back mechanism is maintained. For this reason, the back-driven phenomenon will not occur again. 

**Figure 9:**
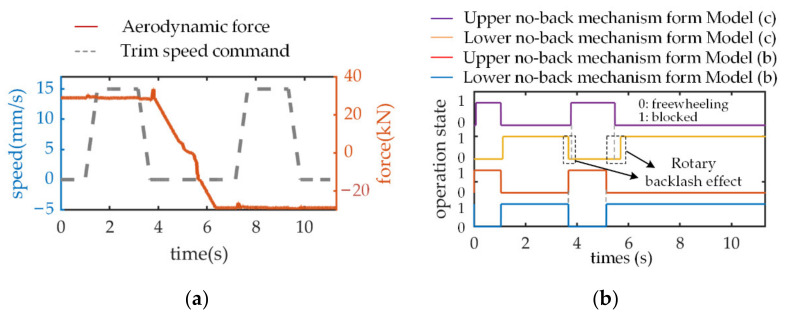

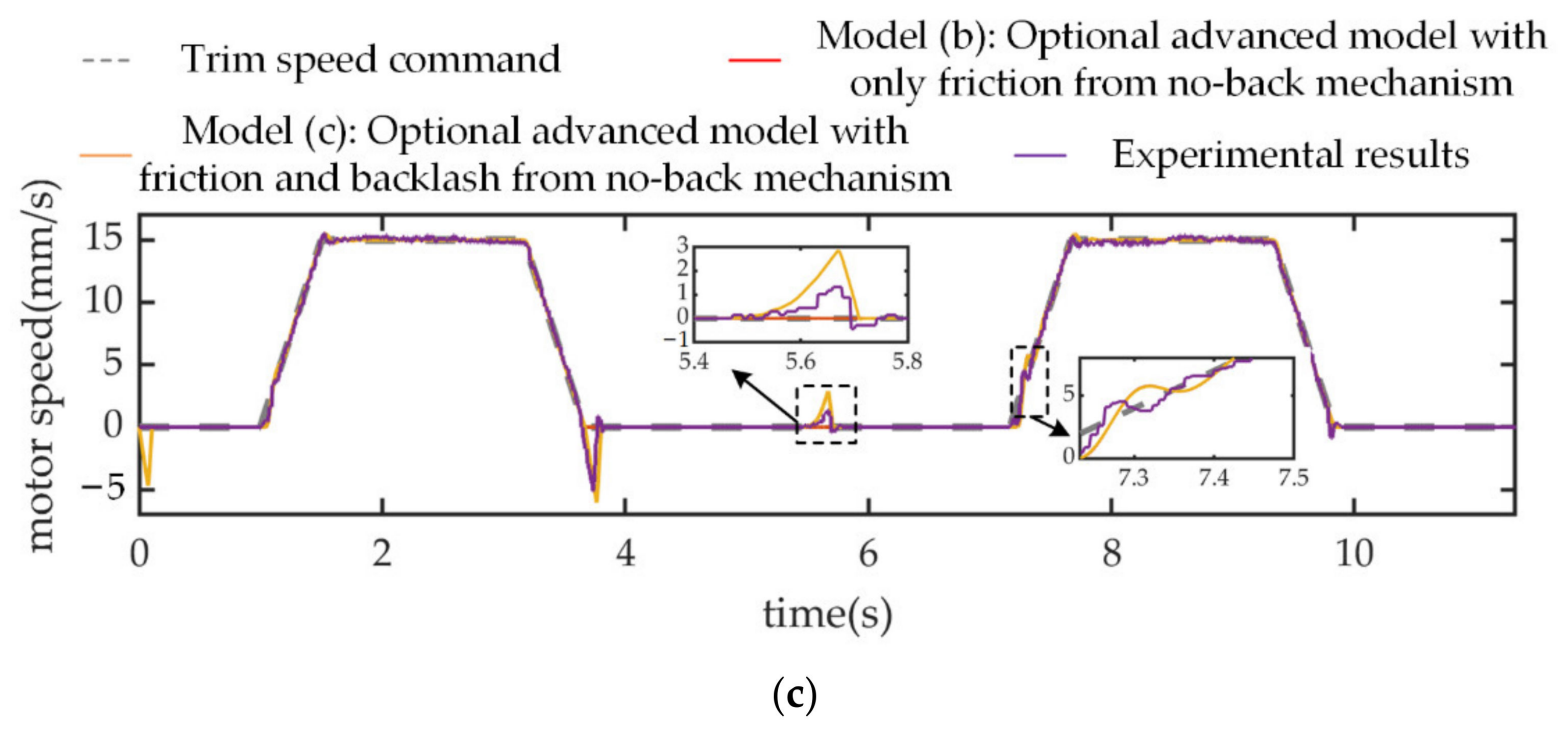
Simulation and experimental results under unipolar speed command and bipolar load. (**a**) No-back friction torque; (**b**) Operation state of pawl and ratchet wheel; (**c**) Motor speed responses from different level models and experimental results.

For further investigation, the speed command signal and aerodynamic force input are redefined as in Figure 9a. During the first trim stop stage, since the compressive load force transforms into the tensile direction, the operation states of both ratchet wheels are inversed again in Figure 9b (approximately time 5.5 s), compared to that in Figure 8b. Compared to model (b), without taking the existence of backlash into consideration, THSA is back-driven by the aerodynamic load in model (c)’s response and the experimental results (Figure 9c), even if the motor is disabled. Meanwhile, when THSA is activated under an aiding load, which leads to the occurrence of relative slip for the lower friction disk, the friction torque will experience a sudden increase. For this reason, the speed response emerges as a speed perturbation, as in Figure 9c. Similarly, these backlash effects are reflected by the time delays of corresponding operation states for pawls and ratchet wheels (Figure 9b).

### 3.2. Nonlinear Compliance Effect of Mechanical Load Path

**Figure 10:**
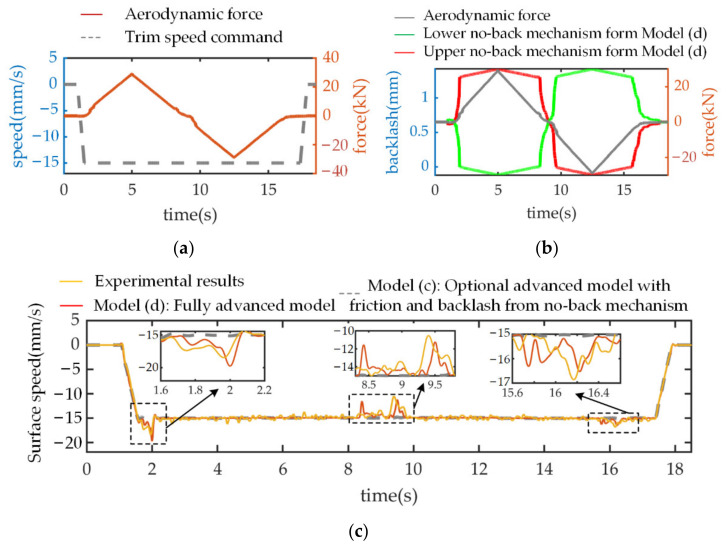
Simulation responses and experimental results under unipolar speed command and triangular load. (**a**) Speed command and load force input; (**b**) Backlash variations versus load force; (**c**) Surface speed responses from different level models and experimental results.

The effect of rotary backlash on system performance is established by comparing the surface speed response of different level models (c,d) and the experimental results. For this purpose, a negative trapezoidal trim speed command with an amplitude of 15 mm/s and acceleration of 30 mm/s^2^ is applied at time 1 s. Simultaneously, a triangular signal with an amplitude of 29 kN and period of 15 s is taken as the aerodynamic force input (Figure 10a).

The clearance variations in the mechanical load path versus the external load for model (d) are depicted in Figure 10b, where the negative magnitude implies that the clearance has been eliminated, i.e., active contact occurs. Since the backlash effect is introduced in model (d), the structural stiffness is mainly dependent on the disk spring, when the aerodynamic force amplitude is not large enough to eliminate the initial clearance. Compared to model (c), with ideal structural stiffness, from Equation (4), this poor stiffness causes an additional linear displacement/speed to the screw–nut mechanism and leads to the oscillation of surface speed shown in Figure 10c. The largest overshoot is around 30 percent of the command speed amplitude. According to Equation (3), when the load force continues to increase, the backlash will be gradually eliminated and the structural stiffness will return to normal. For this reason, the responses from both models and the experimental results are globally consistent within these regions. Since clearances from the upper and lower no-back mechanism are unilateral and there is symmetry about the screw, it is noted that these two clearances show opposite variation trends under the same load direction (Figure 10b).

## 4. Conclusions

This paper takes THSA as an example and focuses on utilizing an incremental approach to progressively introduce individual nonlinear effects into models of different complexity levels. By combining the modelling principles analysis and parameter identification methods, the representative model is used to model the nonlinear effects realistically. Finally, the responses from each model and the experimental results are compared to analyze and verify how nonlinearities affect system performance, with the aim of establishing an approximate complexity model to support the MBSE process.

According to the response comparisons between models of different levels and the experimental results, both the nonlinear compliance effect and friction show significant impacts on system performance. When trimming under aiding load conditions, the significant increase in no-back friction generated from the friction disk leads to approximately three-times larger current demands and a speed perturbation (around 0.1 s) at the activation stage. Before the surface position is mechanically locked by blocking the respective ratchet wheel with corresponding pawls, the no-back rotary backlash must be eliminated first, which causes the screw to be back-driven by the aerodynamic load. Moreover, the maximum back-driven speed can approximately reach 40 percent of the command speed amplitude.

When the aerodynamic force amplitude is not large enough to eliminate the initial clearance of the mechanical load path, the structural stiffness is mainly dependent on the preload disk spring, which causes the oscillation of surface speed and an overshoot with around 30 percent of the command signal. Additionally, the clearances from the upper and lower no-back mechanism are unilateral and symmetrical; for this reason, they show opposite variation trends under the same load direction. 

## Figures and Tables

**Table 1:** Identification results of stiffness parameters.

Parameter	*k*_p,l_ (N/m)	*k*_p,u_ (N/m)	*k*_ds_ (N/m)	*b*_pb_ (mm)
values	6.65 × 10^7^	5.32× 10^7^	1.48 × 10^6^	0.67

**Table 2:** Identification results of friction model parameters.

Parameter	*μ*_s+_ (Nm/N)	*μ*_c+_ (Nm/N)	*f*_s+_ (Nm/(rev/min))	*ω*_st+_ (rev/min)	*μ*_s-_ (Nm/N)	*μ*_c-_ (Nm/N)	*f*_s-_ (Nm/(rev/min))	*ω*_st_ (rev/min)
values	0.0072	0.0048	0	17.62	0.0066	0.0043	0	15.15

**Table 3:** Parameters for model implementation.

Symbol	Item	Value	Symbol	Item	Value
*J*_m_	Inertia of rotor	1.8 × 10^−4^ kg × m^2^	*i*	Total gear ratio	65.61
*R*_s_	Stator resistance	1.23 Ω	*p*	Lead of screw	12 mm
*L*_s_	Stator inductance	2.25 mH	*J*_s_	Screw shaft inertia	0.09 kg × m^2^
*P_s_*	Pole pairs	3	*m*_st_	Load path equivalent mass	80 kg
*Ψ*_f_	Permanent flux linkage	0.072 wb	*m*_hs_	Equivalent mass of surface	350 kg
*I*_max_	Maximum phase current	15 A	*F*_p_	No-back preload force	3000 N
*T*_n_	Motor rated torque	1.9 Nm	*θ*_bmax_	No-back maximum backlash	8.57 degree
*U*_dc_	DC bus voltage of PDE	270 V	*J*_rw_	Ratchet wheel inertia	3 × 10^−3^ kg × m^2^

## Data Availability

The data presented in this study are available on request from the corresponding author.
